# Aromatase inhibitors in post-menopausal endometriosis

**DOI:** 10.1186/1477-7827-9-90

**Published:** 2011-06-21

**Authors:** Nikolaos P Polyzos, Human M Fatemi, Apostolos Zavos, Grigoris Grimbizis, Dimitra Kyrou, Juan-Garcia Velasco, Paul Devroey, Basil Tarlatzis, Evangelos G Papanikolaou

**Affiliations:** 1 OB-GYN University Clinic, University Hospital of Larissa, Larissa, Greece; 2 University Hospital, Dutch speaking Free University of Brussels, Laarbeeklaan 101, 1090, Brussels, Belgium; 3 First Department of Obstetrics and Gynecology, Aristotle University of Thessaloniki, Perifereiaki Odos Thessalonikis-N. Efkarpias 564 29, Thessaloniki, Greece; 4 IVI, Madrid, Avenida Talgo, 68, 28023 Madrid, Spain; 5 Human Reproduction and Genetics Foundation, Adrianoupoleos 6, 55133 Kalamaria, Thessaloniki, Greece

## Abstract

Postmenopausal endometriosis is a rare clinical condition. The diagnosis and treatment of an endometriotic lesion in postmenopausal women is complicated. First line treatment choice should be surgical, given that there is a potential risk of malignancy. Medical treatment may be considered as second line or as an alternate first line treatment whenever surgery is contradicted and aims to alter the hormonal pathway leading to endometriosis progress. Different hormonal regimens have been administered to these patients, with conflicting however results. Aromatase inhibitors (AIs) represent one of the most recently used drugs for postmenopausal endometriosis. Clinical data for the use of (AIs) in postmenopausal patients is scarce. Up to date only 5 case reports are available regarding the use of these agents in postmenopausal women. Although definite conclusions may be premature, AIs appear to considerably improve patients' symptoms and reduce endometriotic lesions size. Nonetheless the subsequent induced reduction in estrogen production, leads to certain short-term and long-term adverse effects. Despite the limited available data, AIs appear to represent a new promising method which may improve symptoms and treat these patients, either as first line treatment, when surgery is contraindicated or as a second line for recurrences following surgical treatment. However, careful monitoring of patients' risk profile and further research regarding long-term effects and side-effects of these agents is essential prior implementing them in everyday clinical practice.

## Review

Endometriosis is a clinical condition mainly seen among women of reproductive age. Nonetheless, it may also affect post-menopausal women [1], with a prevalence varying from 2 to 5% [2]. In 1942 Haydon reported a 78-years-old patient with endometriosis, [3], whereas in another study 138 endometriosis patients were reported to be 2 or more years post-menopausal [4]. Aromatase inhibitors (AIs) represent one of the most recently used drugs for postmenopausal endometriosis. They were first used for the treatment of postmenopausal, estrogen receptor positive advanced breast cancer due to their ability to reduce estrogen production through inhibition of cytochrome P450.

### Pathogenesis of postmenopausal endometriosis

Endometriosis is always estrogen dependent. While in premenopausal women the ovaries are the main source of estrogen production, in postmenopausal patients estrogens are derived either from exogenous administration, or from endogenous extraovarian production. Several reports have linked postmenopausal endometriosis with hormone therapy [5,6]. However, estrogen production during menopause may be derived from extra-ovarian sources such as the adrenal glands, the skin, the endometrial stroma, and the adipose tissue [7], with the latter probably accounting the larger part of estrogen production through aromatation of adrenal gland androgen [8].

### Treatment options for patients with postmenopausal endometriosis

First line treatment for endometriosis in postmenopausal patients should be surgical. The rationale behind such an approach is that any postmenopausal mass has a potential risk of malignancy and therefore should be removed [9]. On the other hand, the risk of malignant transformation of endometriosis may be increased[10]. However, despite the fact that surgical approach should be the first line treatment, recurrence rates after surgery are increased [11,12]and therefore an effective second line treatment for these patients is mandatory. Furthermore, given that operation may not always be feasible, an alternative first line treatment should be considered.

Medical treatment aims to alter the hormonal pathway leading to endometriosis progress. The use of GnRH agonists, progestins or danazol, intend through a step-wise pathway to decrease estrogen production, or alter the estrogenic effect. Yet, considering that treatment with either GnRH agonists [7]or progestins appears be ineffective in postmenopausal patients [11], the need for alternative drugs to reduce estrogen production is mandatory.

AIs may be considered an efficient treatment modality for these patients since, compared to other hormonal therapies such as GnRH agonists, they have the ability to further block extraovarian estrogen production which is the main estrogen source for these women.

### Aromatase inhibitors (AIs)

Aromatase Inhibitors were first used for the treatment of postmenopausal, estrogen receptor positive advanced breast cancer [13]. Their ability to reduce estrogen production is through inhibition of cytochrome P450, a key enzyme that catalyzes conversion of andostenendione and testosterone to estrone and estradiol[14].

Letrozole and anastrozole are triazole derivatives that are reversible, competitive AIs and, at doses of 1-5 mg/day, inhibit estrogen levels by 97% to more than 99% [15], whereas exemestane is a steroidal, irreversible inhibitor that binds to the active site of the aromatase enzyme and inactivates it effectively at a dose of 25 mg/day. AIs may offer a new alternative for postmenopausal patients with endometriosis through the alteration of mechanisms involved in molecular development of endometriosis [16].

Evidence regarding the use of AIs in premenopausal patients is far more extensive compared to postmenopausal women, mainly due to the considerable difference in the prevalence of disease among these groups of patients. Nonetheless, it appears that AIs in premenopausal endometriosis, represent only a part of the therapeutic plan. Previous reports have raised arguments regarding the proposed beneficial effect of AIs in these women, suggesting that this may be attributed to the combined use with other agents (e.g. GnRH agonists, danazol, oral contraceptives (OCs), progestins) [17]. The main reason for these objections is the fact that in premenopausal women the main source of estrogens is the ovary. Consequently, premenopausal endometriosis is often successfully suppressed by estrogen deprivation with GnRH analogs or the induction of surgical menopause [18]. Therefore, in there women AIs may only be justified when GnRH analogs fail to control the disease through the elimination of estradiol secreted by the ovary [18], probably due to the presence of significant estradiol production that continues in the adipose tissue, skin, and endometriotic implant per se during the GnRH agonist treatment.

On the contrary, in postmenopausal women the only estrogen activity is related to the production from extraovarian sources and consequently AIs appear to be effective in treating severe endometriosis through blockade of extraovarian estrogen production. This is the key for the efficacy of AIs in the management of hormone receptor positive breast cancer. At the moment 3^rd ^generation of AIs are used for the treatment of postmenopausal breast cancer patients [18]. Hence, given that they successfully control breast cancer disease in postmenopausal women though the reduction in circulating estrogens [13], it is likely that they may also have effect in postmenopausal endometriosis. Blockage of aromatase activity derived from extraovarian sites with an aromatase inhibitor may keep a larger number of patients in remission for longer periods of time. Furthermore, the effect of AIs in postmenopausal endometriosis may even be greater given that they are involved in the pathologic pathway within the endometriotic lesions. An intracrine mechanism production of large quantities of estrogen has been proposed within the ectopic tissue endometriotic cells. Endometriotic implants although histologically similar to eutopic endometrium, appear to be different in their molecular base and this may give rise to extreme production and impaired metabolism of estradiol [8,11,18,19]. Therefore, AIs can theoretically markedly reduce this production and thus decrease the size of the lesions.

### Available evidence regarding the use of AIs for the treatment of postmenopausal endometriosis

Although a prospective randomized trial showed that AIs in combination with GnRH analogues increased the pain-free interval and decreased symptoms recurrence rates following surgery in premenopausal patients with severe endometriosis [20], clinical data for the use of **(AIs) **in postmenopausal patients is limited.

Up to date only 5 case reports are available regarding the use of AIs in postmenopausal women and thus definite conclusions may be premature (table 1). Patients' age ranged from 47 to 61 years old. The majority of them had total abdominal hysterectomy and bilateral oophorectomy at earlier age. Two patients had received hormonal replacement therapy during menopause, one until recurrence of endometriosis [11]and one more than 3 years prior recurrence[7]. The majority of women had been previously treated for endometriosis with either surgery, or GnRH agonists or progestins. Finally, all cases involved patients with either surgical [7,11,21,22] or natural [23]menopause.

**Table 1:** Studies regarding the use of aromatase inhibitors for postmenopausal endometriosis

Study (year)	Study type	Age	Menopause	Use of HRT during menopause	Clinical manifestation	Previous interventions fendometriosis treatment	Interventions	Treatment in months	Outcome			Supplementation for osteoporosis	Side effects	
									Pain	Lesion size reduction	Other outcomes			
Takayama *et al*. (1998) [11]	Case Report	57	Surgical	Yes	Polypoid mass at the vaginal apex (recurrence)	Surgery Megestrol acetate	Anastrozole 1 mg	9 months	Pain relief after 1 month-discontinue of pain medication	Size before therapy 30 × 30 × 20 after 6 months 6 × 3 × 5.	Change in mass color from red to pale gray.	Alendronate (1 mg/d) for 9 months, calcium (1.5 g/d) and vitamin D (800 U/d)	Reduction in bone mineral density (mild)	lumbar spine-6.2%, femoral neck-3.7%
Fatemi *et al *(2005) [7]	Case Report	55	Surgical	Yes	Mass 4 × 8 cm (recurrence)	Surgery GnRH agonist	Letrozole 2.5 mg	18 months	Started to reduce after 3 months, At 12 months completely asymptomatic	Size before therapy 4 × 8 3 months no reduction 6^th ^month 6 × 5 18^th ^month 1 cm	No active capitation of contrast during CT scan (18 months)	Alendronate (70 mg/week) Vit D (440/day), calcium (1,2 g/day)	No	
Mousa et al (2007) [21]	Case Report	Middle-aged	Unclear	No	Endometriotic nodule in bladder wall (recurrence)	No	Exemestane 25 mg	15 days	No improvement	NA	NA	No	No	
							Letrozole 2.5 mg	4 months	Pain relief Urinary tract symptoms relieved	NA	NA	No	Hot flushes	
							Letrozole 2.5 mg	4 months	Pain relief Urinary tract symptoms relieved	NA	NA	No	No	
Bohrer et al (2008) [22]	Case Report	47	Surgical	No	Ureteral endometriosis Bowel endometriosis (recurrence)	Surgery Megestrol acetate GnRH agonist	Anastrazole 1 mg	15 months	Pain relief Bowel symptoms resolved	NA	NA	No	NA	
Sasson et al (2009) [23]	Case Report	61	Natural	No	Mass 12.5 × 8 cm (recurrence)	Surgery	Letrozole 5 m +MPA 10 mg after the 3^rd ^wk (repeated aspirations	4 months	NA	Size before 12.7 × 8.2 × 10.0 cm 2^nd ^week, minimal change 4th month size 6.6 × 8.5 × 4.2 cm	NA	No	NA	

#### AIs and symptoms relief in postmenopausal patients

In all treated patients, administration of letrozole or anastrozole appeared to improve pain related to endometriosis, either when treatment was administered for 4 months or for up to 15 months. Furthermore, letrozole appeared to improve all the other symptoms, such as urinary tract and bowel symptoms, whenever these systems were affected by endometriosis. Nonetheless, exemestane did not improve endometriosis symptoms in one patient; when in the same patient letrozole was subsequently administered, a significant beneficial effect on symptoms relief was observed. Although one may interpret this observation as a potential difference between these two agents' efficacy, this should be interpreted with caution, given the short term of administration of exemestane [21].

Besides subjective improvement, related to patients' reported symptoms, letrozole or anastrozole has shown a beneficial effect in quantitative parameters such as the endometriotic lesions size. In all of the patients in which clinical manifestation of the disease was the presence of endometriotic masses, AIs significantly reduced the size of the lesions as measured by imaging techniques.

The only symptom that appeared not to improve even 15 months after the administration of anastrozole was ureteral endometriosis. However this patient suffered from extensive ureteral fibrosis. Thus, a potential explanation for this lack of effect of treatment may be the inability of these agents to improve ureteral obstruction, since fibrosis does not respond to hormonal therapy [24,25].

#### Side effects of AIs-how can they be minimized in patients with endometriosis

Despite the fact that AIs appear to considerably improve patients' symptoms and reduce endometriotic lesions size, their use and the subsequent induced reduction in estrogen production, leads to certain short-term and long-term adverse effects. Hot flushes, vaginal dryness, arthralgias, decreased bone mineral density, have been reported [26]. However, the most important risk associated with AIs administration is osteoporosis and an increased fracture rate [26]. Long term use of AIs in the adjuvant setting has been associated with a significantly higher bone fracture rate compared to tamoxifen especially in patients with advanced age, smoking history, osteoporosis at baseline, previous bone fracture, and previous hormonal replacement therapy[27].

Among the patients treated up to date with AIs for postmenopausal endometriosis, only one reported hot flushes after 4 months of letrozole administration, as side-effect of medication; co-administration of micronized estradiol 0,5 mg daily improved hot flushes and no pain recurrence occurred over the following 4 months.

To reduce the risk of osteoporosis in high-risk patients, bisphosphonates may be co-administered with AIs during long-term treatment. For breast cancer patients the American Society of Clinical Oncologists recommends that bone mineral density screening should be repeated annually in all patients receiving aromatase inhibitor adjuvant therapy, and bisphosphonate therapy should be initiated when the Bone Mineral Density Measurement which is commonly reported in terms of T-score, are -2.5 or lower [28]. Among the available studies, co-administration of biphosphonates (aledronate) was given in two patients; still one of them reported letrozole associated bone loss with bone mineral density marginally reduced following 9 months treatment with 1 mg anastrozole.

## Conclusions

Postmenopausal endometriosis is a rare clinical condition which may significantly impair patients' quality of life. Despite the considerably limited available data, AIs may be a new promising method which could potentially improve symptoms and treat these patients, either as first line treatment, when surgery is contraindicated or as a second line for recurrences following surgical treatment. The crucial however question remains whether such an approach has the potential to be an effective long term treatment, given that current reports involve few patients with a limited follow-up. Furthermore another serious issue is whether AIs truly is a cost-benefit method. Taking into account that treatment with AIs may significantly impair bone mineral density and increase the rate of bone fractures, it is intriguing to clarify whether the benefits gained from this treatment modality outnumber the harms in patients with postmenopausal endometriosis. Furthermore, prior administrating these agents in patients with postmenopausal endometriosis considering patient's risk profile may be the most stepwise approach.

## List of abbreviations

AIs: Aromatase inhibitors; MPA: medroxyprogesterone

## Competing interests

The authors declare that they have no competing interests.

## Authors' contributions

NPP wrote the manuscript, HMF wrote the manuscript, AZ, wrote the manuscript, GG wrote the manuscript, DK, reviewed the literature, JGV, revised the manuscript, PD revised the manuscript, BT revised the manuscript, EGP revised the manuscript. All authors read and approved the final manuscript.
